# The Problem of Cancer

**Published:** 1924-10

**Authors:** John Hall-Edwards

**Affiliations:** Major (late R.A.M.C.)


### THE PROBLEM OF CANCER.

### A PLEA FOR IMMEDIATE ACTION.

By JOHN HALL-EDWARDS, L.R.C.P. Ed., D.R.M.E. Cantab., F.R.S. Ed., Major (late R.A.M.C.).

Cancer is largely a disease of ignorance, and the only cure for ignorance is trustworthy information. In America, through the instrumentality of the Society for the Control of Cancer, the object of which is the dissemination of knowledge concerning its symptoms, diagnosis, treatment and prevention, much excellent propaganda work has been carried out. It has been acknowledged by Hoffman (the distinguished statistician and authority upon Cancer) that this intensified propaganda has unquestionably diminished the death rate and has proved that Cancer Control is being realized to an increasing extent.

### "Cancer Week" in America.

Under the auspices of the American Society for the Control of Cancer, "Cancer Weeks" have been organised, during which an intensive campaign has been carried on in the newspapers and by addresses in churches and theatres, by posters, and films in picture houses. Ten million people are supposed to have learned the vital facts of Cancer control during the period, and it is stated on the best authority that no special alarm resulted from the campaign, while much good accrued. As the result of articles I have recently written on this subject, I have received much correspondence from interested readers who, while agreeing with me that everything possible should be done to combat the disease, say that on no account must anything be said or done which is likely to produce alarm in the mind of the public.

### The Change in the Public Mind.

The public mind has, however, undergone consider able alteration during the last few years, and I think that at the present time, taking into consideration the ever-increasing death rate, the public mind is more likely to be assuaged than alarmed by trustworthy information. Even were this not the case the whole question is so grave that I have every confidence in saying that we should not be deterred from undertaking an obvious duty in the face of the small amount of alarm which might ensue. It is almost unnecessary in these columns to state that neither education in control nor prevention is likely to obtain the same results as have been achieved by the treatment of diseases in which the cause is well recognised. Only a percentage of lives can be saved.

### Empire Propaganda.

Public Health Authorities have it in their power to deal out information on the subject, through the instrumentality of the general practitioners, the nurses, welfare workers and teachers under their control, and I have before suggested that every such authority should appoint a Cancer sub-committee with this object. This has already been done in some towns and has met with the unequivocal approval of the Minister of Health. Some years ago the Borough of Portsmouth issued a circular in regard to Cancer, based entirely upon the lines of Cancer Control. What effect this had it is difficult to ascertain; other towns have made similar attempts. This, in my opinion, is of little use. I feel that if any lasting good is to be obtained such propaganda work must be undertaken throughout the whole of the Empire, and the time has arrived when, so far as this country is concerned, the Ministry of Health could order this to be done, and would be justified in doing so. Public apathy with regard to Cancer is largely due to the fact that in the early stages of the disease there is frequently little or no pain. This apathy can only be combated by the public being urged to seek medical advice when—in certain forms of the disease —premonitory symptoms present themselves, and drawing attention to the fact that by certain precautions the inception of the disease can be prevented.

### Attitude of the Royal Society of Medicine.

Up to quite recent times the bogey of alarm had such a hold upon the medical profession that many declined to entertain the idea of public education on the subject, but in March last year, before the Royal Society of Medicine, Mr. E. T. Adams read a paper on "The urgent need for education in the Control of Cancer." Lord Dawson expressed the opinion that "education should begin with the medical profession; he was afraid of alarming the public by a campaign on American lines." Sir Napier Burnett suggested the giving of public lectures on "How to keep well," and as the outcome of this meeting the following resolutions were carried: "1. That it is desirable that the public should be given more information as to the early signs of Cancer, and the prospects of cure by immediate treatment. 2. That the British Red Cross Society be asked to conduct this publicity campaign by means of lectures and pamphlets. 3. That the Council of the Royal Society of Medicine be requested by this meeting to nominate a standing committee to supply information to the British Red Cross Society suitable for wide dissemination and the education of the public."

### Need for an Intensified Campaign.

The passing of these resolutions by a Society of such importance as the Royal Society of Medicine is proof positive that the medical profession is now aware of the necessity of public education in these matters. These resolutions have paved the way for the carrying out of the ideas I have in mind, and nothing but apathy now stands in the way of prosecuting these ideas. No further words of mine should be necessary to impress upon my readers the fact that an intensified campaign against Cancer is urgently needed. The time has now arrived when England should wake up and "strike the iron while it is hot." In suggesting such a campaign it is far from my wish to place any hindrance in the way of scientific research into the many problems of the disease. The two campaigns can work hand in hand to their mutual benefit.

